# Hyperprolactinemia Associated with Attentional Processing and Interference Control Impairments in Patients with Prolactinomas

**DOI:** 10.3390/brainsci12081091

**Published:** 2022-08-17

**Authors:** Aobo Chen, Chenglong Cao, Bangxin Liu, Shuochen Wang, Shukai Wu, Guozheng Xu, Jian Song

**Affiliations:** 1 The First School of Clinical Medicine, Southern Medical University, Guangzhou 510515, China; 2 Department of Neurosurgery, The General Hospital of Chinese PLA Central Theater Command, Wuhan 430074, China; 3 Department of Cognitive Neuroscience, Faculty of Psychology & Neuroscience, Maastricht University, P.O. Box 616, 6200 MD Maastricht, The Netherlands

**Keywords:** prolactinoma, event-related potentials, attention, interference control, cognition

## Abstract

The cognitive impairment of pituitary adenomas (PAs) has received increasing attention. Hyperprolactinemia and tumor mass effect are the potential causes. The aim of this study was to identify possible cognitive impairment and to further explore the correlation between these indices and prolactin (PRL) levels, based on the control of tumor size. Twenty-seven patients with prolactinomas (patient group) and twenty-six matched health control group (HC group) were enrolled in this study. All participants performed the flanker task while we continuously recorded electroencephalography data. On the behavioral performance level, patients showed a significantly slower reaction time (RT) in both flanker types. Concerning the event-related potentials level, patients elicited reduced P2 and enhanced N2 amplitudes compared with the HC group, suggesting an impairment of attentional processing (P2) and conflict monitoring (N2). Moreover, the patient group also induced lower P3 amplitudes relative to the HC group in both types, indicating that there were deficits in attentional resource allocation ability. We also found a significant correlation between the P3 amplitudes and incongruent condition RTs, as well as the subsequent PRL levels in the patient group. In conclusion, this is an innovative study that reveals the impaired cognition abilities in prolactinomas, and also proposes the possible cognitive toxicity of oversecreted PRL levels, which provides evidence for further research on the cognitive decline in PAs.

## 1. Introduction

Pituitary adenomas (PAs) are benign, slow-growing, and monoclonal central nervous system (CNS) tumors (except for pituitary adenocarcinoma), with a prevalence second only to meningiomas, at approximately 80–90 cases per 100,000 [1]. According to the type and size, PAs can be comprehensively managed with surgery, radiotherapy, and medication. In recent years, the application of neuroendoscopy, endocrine management, and other comprehensive treatment methods has enabled better early intervention; it has also allowed for a more likely total resection, which has significantly improved the prognosis and quality of life of patients with PAs [2,3]. However, emerging evidence has revealed varying degrees of cognitive impairment in patients with PAs [4,5,6,7,8].

In 1992, Grattan-Smith et al. [9] conducted the first relevant study, pointing out that neuropsychological changes were very commonly seen in patients with PAs and thus need clinical attention. After this study, Meyers et al. [10] systematically reported that for the first time, the incidence of cognitive dysfunction in PAs was as high as 50%. The convenience and operability of the neuropsychological scales led to a series of subsequent studies, while the lack of restrictions on potential confounding factors such as tumor size, tumor subtypes, and treatment methods served as a reason for the controversial conclusion [11]. Some studies have revealed the impairment of cognitive function in patients with hormone-secreting PAs and concluded that the over-productive hormones have possible cognition toxicity [12,13,14]. Specifically, decreased scores on neuropsychological tests have been reported in active Cushing disease (CD) patients, prevalent in the form of memory and psychiatric symptoms [15]. Acromegaly patients with deficits in attention, executive, and decision-making abilities were associated with long-term exposure to high levels of growth hormone and IGF-1, where the severity of this exposure is proportional to the hormone concentration [16]. As for the subtype that secretes prolactin (PRL), a case report described a huge PRL-secreting adenoma with the symptoms of dementia, which disappeared after a bromocriptine treatment [17]. However, a similar pattern of cognitive impairment was also revealed in non-functioning PAs (NFPAs). Butterbrod et al. [18] found that 45 patients with NFPAs also showed cognitive deficits that could only be associated with the tumor size. To date, it remains unclear whether these presentations were the results of hormone secretion [19], local tumor pressure [20], or a combination of both. Therefore, further research needs to control the above potential variables in the design. 

Based on the above considerations, the current study selected patients with prolactinomas for the following reasons: (1) The incidence of prolactinoma is the highest among all pituitary adenoma subtypes, which facilitates the recruitment of suitable subjects, (2) Literature review suggested that cognitive impairment caused by hyperprolactinemia was relatively more reported, (3) The clinical symptoms caused by hyperprolactinemia are easier to be detected early compared with other subtypes, so it would be easier to recruit small tumors that meet the criterion.

Prolactinoma is the most common subtype of PAs (accounting for up to 60%), of which the main clinical damages are attributed to tumor local mass effects and prolactin oversecretion [21]. Bala et al. [4] recruited 20 prolactinoma patients and 20 healthy control participants in a case-control study comparing cognitive function using a comprehensive battery of neuropsychological tests; the authors discovered a significant relationship between PRL overproduction and a worsening of cognitive processes, especially in memory and attention. Individuals in a state of hyperprolactinemia have dopamine inhibition, which may be a key path leading to cognitive decline [22]. Notably, a report introduced one patient with an elevated PRL level due to a craniopharyngiomas compression of the pituitary gland; this individual exhibited similar deficits in attention, short-term memory, naming, and visual spatial functionality [23]. Moreover, patients with prolactinomas often complain of dysfunction in their cognitive control function. Cognitive control, also known as executive control, is a high-level cognitive process defined as the regulation of the executive functions on the integration of incoming information, perception, and response execution [24]. This process mainly includes attention, goal maintenance, switching, working memory, inhibition, etc. [25]. Voxel-based morphometry analysis manifested a decreased gray matter volume (GMV) in the right middle frontal cortex (MFC) and right inferior frontal cortex (IFC) in prolactinoma patients, and these areas are associated with inhibition processes [26]. Therefore, this study mainly investigated the altered inhibition ability of prolactinoma patients.

Interference control, one of the two types of inhibition ability (the other is response inhibition), refers to the ability to maintain focus on relevant responses, tasks, or stimuli in an environment that is being disturbed by task-related information. The Eriksen flanker task [27] was one of the most investigated experimental paradigms and was used to study conflict resolution and interference control; the modified arrow version was employed in this study. This paradigm consists of a central target arrow that is surrounded by flanker arrows that either point to the same direction (congruent) or opposite direction (incongruent) as the central arrow. When participants were instructed to identify the target arrow direction, incongruent conditions usually lead to longer reaction times and lower response accuracy than in congruent conditions. The process of focusing on the central arrow and ignoring flanker arrows indexed the interference control aspects of inhibition.

Event-related potentials (ERPs) is a technology that has been popularized in cognitive neuroscience in the recent years. The technology has provided us with further insights to study the synchronized neuronal activity processes related to specific events with a high temporal resolution [28]. Previous ERP studies using the Eriksen flanker task (see review [29]) have already indicated that the N2 and P3 components reflected inhibition control. The N2 component is an early negative waveform that appears approximately 200–300 ms (milliseconds) after the stimulus onset and is distributed in the frontal–central regions. It is derived from the anterior cingulate cortex (ACC) and serves as an indicator of interference control [30]. Previous evidence has suggested that N2 represents the pre-response conflict elicited by the activation of the target arrow and flanking arrows. Thus, the N2 component is related to the conflict monitoring process before the task completion [31]. The P3 component is a later positive waveform that appears approximately 300–500 ms after the stimulus onset and is distributed in midline regions [32,33]. Neuhaus et al. [34] demonstrated that the midline parietal P3 amplitudes were associated with the executive control process. Therefore, the N2 and P3 components were regarded as important neurophysiological markers in the present study. Additionally, another ERP component must be mentioned here, which was not often investigated in other flanker studies. P2, an early positive-going component with a peak latency ranging from 150 to 250 ms after the stimuli onset, is considered to reflect an early allocation of attention and awareness. In several emotional-related studies, P2 has also been implicated in emotional processing, and is especially sensitive to negative stimuli-like threatening images [35]. In the current study, P2 appeared to be another relevant component in the exploration of possible cognitive changes under conflicting conditions. 

To the best of our knowledge, there is currently no research on the deficits in interference control in patients with prolactinomas. Additionally, this paper intends to further explore the potential correlations between cognitive indices (behavioral level and neurophysiological level) and prolactin hormone levels by adopting strict screening criteria to control the confounding factors such as tumor mass effect as much as possible. We hypothesized that the patient group would have similar behavioral results to the healthy control group, with significant differences in ERP indices. Moreover, certain correlations would be revealed between the PRL level and ERP indices. That is, a higher PRL level would be correlated with worse cognitive function.

## 2. Materials and Methods

### 2.1. Participants

From July 2020 to August 2021, 30 prolactinoma patients were screened in the Department of Neurosurgery, at the Wuhan School of Clinical Medicine, Southern Medical University (China). Meanwhile, 27 healthy control (HC) group members were recruited through advertisements in our hospital, the adjacent university, and other public areas. The inclusion criteria of patients were as follows: (1) Tumor size: microadenoma (< 10 mm in diameter), or macroadenoma (20 mm > diameter > 10 mm), but no obvious invasion of bilateral cavernous sinuses or optic nerve displacement; (2) Tumor type: prolactin-secreting adenoma; (3) Age: ranging from 18 to 55 years old; (4) Education background: more than 9 years; (5) Vision: normal or correct-to-normal vision acuity without a field defect; and (6) Therapy: no therapy history of dopamine agonist drugs. The inclusion criteria of HCs were: (1) Age: ranging from 18 to 55 years old; (2) Education background: more than 9 years of schooling; and (3) Vision: normal or correct-to-normal vision acuity. Both patients and HCs had to be excluded if they: (1) Had a craniotomy or radiation therapy history; (2) Had a neurological or history of psychiatric disorders; (3) Were post-menopausal female participants; (4) Had taken psychotropic drugs and contraceptives in the past 3 months or abused alcohol; or (5) Had other diseases or complications that could affect cognitive function. The study was approved by the ethics committee of the General Hospital of PLA Central Theater Command, and informed consent was fully explained before the experiment and was obtained from all participants.

### 2.2. Stimuli and Procedure

**Figure 1:**
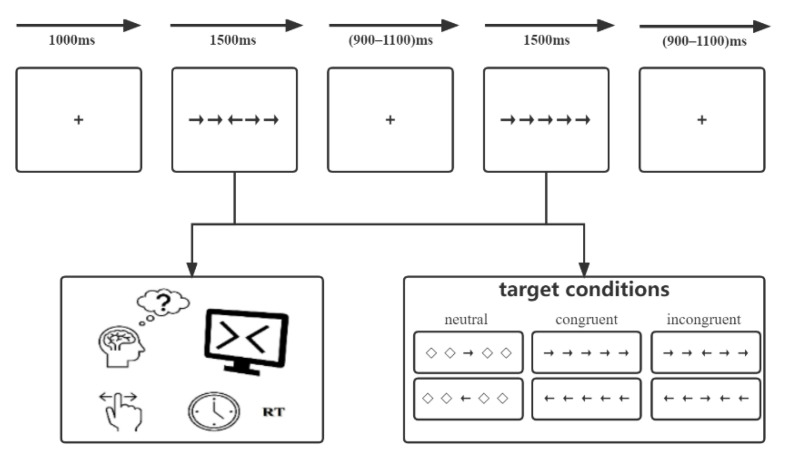
The modified Eriksen flanker task paradigm.

We introduced a modified Eriksen flanker conflict task for all participants. The task was performed using E-prime 2.0 software (Psychology Software Tools, Inc., Sharpsburg, PA, USA) on a computer with a 17-inch Dell monitor. A semi-dark, quiet room was prepared, and participants were instructed to sit and face the monitor, which was placed 80 cm from their eyes. The monitor background was white, and stimuli were black. As shown in Figure 1, the modified flanker paradigm mainly includes five horizontally arrayed arrows with different directions, which formed three types of stimuli: (1) neutral: the central arrow was flanked by a diamond. (2) congruent: all arrows were pointed in the same direction. (3) incongruent: the central arrow pointed in the direction opposite to that of other arrows. Each trial began with the presentation of a fixation cross (0.5 × 0.5 cm, 0.5° angle) centered on the screen with a fixed duration of 1000 ms. Afterwards, the stimuli were presented for 1500 ms at the same location. Participants were instructed to make a response to the direction of the central arrow correctly and quickly by clicking the left/right mouse button. After that, a random interval ranging from 900 to 1100 ms of the fixation was set to reduce the potential expectancy. For each participant, there was a practice portion before the experimental part. Thirty trials were designed to achieve an exceeded 95% accuracy, while feedback of “correct!” or “incorrect!” was presented on the screen. The experimental portion consisted of 180 trials total and contained 3 blocks, and each block contained 60 trials without feedback. Moreover, three types of stimuli were presented equally within each block. Participants were instructed to stay relaxed, concentrate on the center of the screen, and minimize blinking and unnecessary movements as much as possible.

### 2.3. EEG Acquisition

Continuous EEG data were transformed and recorded using a 32-channel array elastic cap with Ag/AgCl electrodes via an eegoTM amplifier (ANT Neuro Inc., Hengelo, The Netherlands). The distribution of electrodes referred to an extended 10/20 international system. The online recording was referred to CPz and continuously digitized at a sampling rate of 1000 Hz and with a 0.3–30 Hz (24 dB/octave; zero phase) bandpass. All electrodes’ impedances were kept below 5 KΩ during the acquisition.

### 2.4. Behavioral Data Analysis

The average RTs and accuracy were calculated by a 2 (group: patients, HCs) × 2 (type: congruent, incongruent) repeated measures ANOVAs were calculated. Response time exceeding 1000 ms or less than 200 ms or incorrect trials were excluded since the anticipation and delayed responses. The group variable was a between-subject factor and other variables were within-subject factors.

### 2.5. Electroencephalography Data Analysis

All participants’ EEG data were analyzed offline using the EEGLAB toolbox, based on MATLAB (MathWorks Inc., MA, USA). For preprocessing, we conducted a 0.01–30 Hz bandpass and a 50 Hz notch filter. Epochs were defined relative to the onset time of stimuli. All EEG data were segmented into 800 ms lengths from 200 ms pre-stimulus to 600 ms post-stimulus, and the baseline was corrected according to the mean amplitude of 200 ms pre-stimulus. The independent component analysis (ICA) method was conducted to identify the vertical and horizontal ocular, muscle, and heat activity artifacts, and these artifacts were removed from the signal. EEG data were further re-referenced to the average of bilateral mastoid (M1, M2) sites. Epochs exceeding ± 70 μV were rejected for further analysis and were identified by visual inspection. EEG data were separately averaged for each participant.

All ERP components were analyzed based on a visual inspection of the grand-average waveforms. Based on the previous hypothesis and the inspection, we identified the P2, N2, and P3 components for analysis. P2 is mainly distributed in the frontal region (FPz, Fz, Cz), while N2 and P3 are mainly distributed in the frontal and central regions (Fz, FCz, Cz, CPz). For both groups, P2 and P3 were calculated for mean amplitudes and N2 was calculated for peak amplitudes due to the ERP components’ pattern. The N2 peak amplitude was found in the 200–300 ms time window. The P2 and P3 mean amplitudes were calculated in the 50 ms and 150 ms time window around the ERP peak point, respectively. In the above analyses, the group variable was defined as a between-subject factor and other variables were defined as within-subject factors. We calculated 2 (group) × 2 (type) × 3/4 (site, P2 [FPz, Fz, Cz], N2 [Fz, FCz, Cz], P3 [Fz, FCz, Cz, CPz]) repeated measure ANOVAs. All effects with more than one degree of freedom were adjusted for sphericity violations using the Greenhouse–Geisser correction. Moreover, the Kolmogorov–Smirnov statistical analysis was used to verify that the PRL level, behavioral performance (accuracy, RT), and ERP amplitudes approximated a normal distribution (all ps > 0.05). Based on the above results, a Pearson correlation analysis was conducted to explore the correlations between the PRL level (ng/mL) and cognitive indices.

## 3. Results

A summary of the demographic characteristics of the participants in both groups is listed in Table 1. During the EEG data preprocessing, 4 participants (3 patients and 1 HC) were excluded from the study due to excessive signal noise in the EEG data.

### 3.1. Behavioral Results

***Accuracy*.** For the flanker effect, the ANOVAs did not reveal a significant main group effect and group × type interaction effect. Group effect was F (1,51) = 0.477, *p* = 0.493,$\;\eta_{p}^{\;2}$ = 0.009; group × type, F (1,51) = 0.027, *p* = 0.871, $\eta_{p}^{\;2}$ = 0.001, but the ANOVA revealed a main type effect, where F (1,51) = 4.319, *p* = 0.043, $\eta_{p}^{\;2}$ = 0.078. For both groups, participants showed more errors in incongruent trials than in congruent trials (98.67% vs. 99.45%).

***RT*.** The ANOVA analysis of the flanker effect revealed a main group effect, where F (1,51) = 11.357, *p* = 0.001, $\eta_{p}^{\;2}$ = 0.182, with the HC group (M = 539.023 ms) showing a significantly shorter RT than the patient group (M = 602.119 ms). The main type effect [F (1,51) = 185.511, *p* < 0.001, $\eta_{p}^{\;2}$ = 0.784] and the interaction of group × type [F (1,51) = 8.752, *p* = 0.005, $\eta_{p}^{\;2}$ = 0.146] were significantly. Post hoc analysis showed that regardless of congruent or incongruent conditions, the HC group responded significantly more quickly than that of the patients (congruent, M = 504.017 ms vs. 575.376 ms, *p* = 0.007; incongruent, M = 553.611 ms vs. 652.492 ms, *p* < 0.001).

### 3.2. Electrophysiology Results

**Figure 2:**
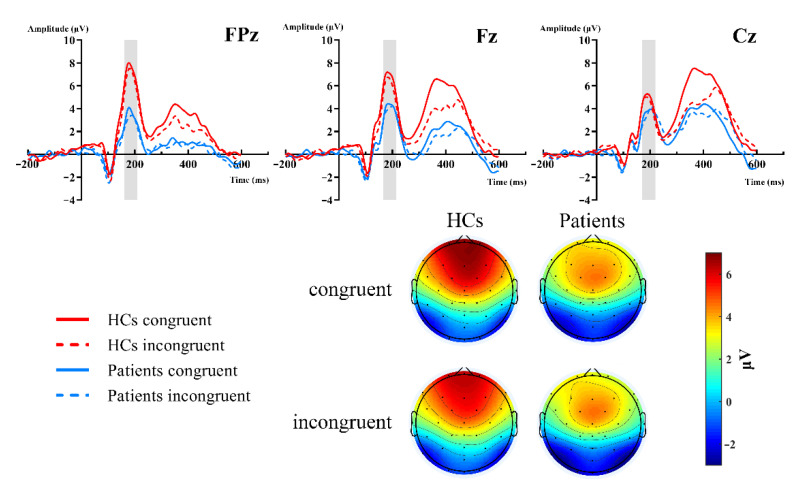
Grand-averaged ERP of the P2 waveforms and topographic voltage maps, comparison of the FPz, Fz, and Cz sites by group. HC group: red lines; patient group: blue lines; congruent trials: solid lines; incongruent trials: dashed lines.

**Figure 3:**
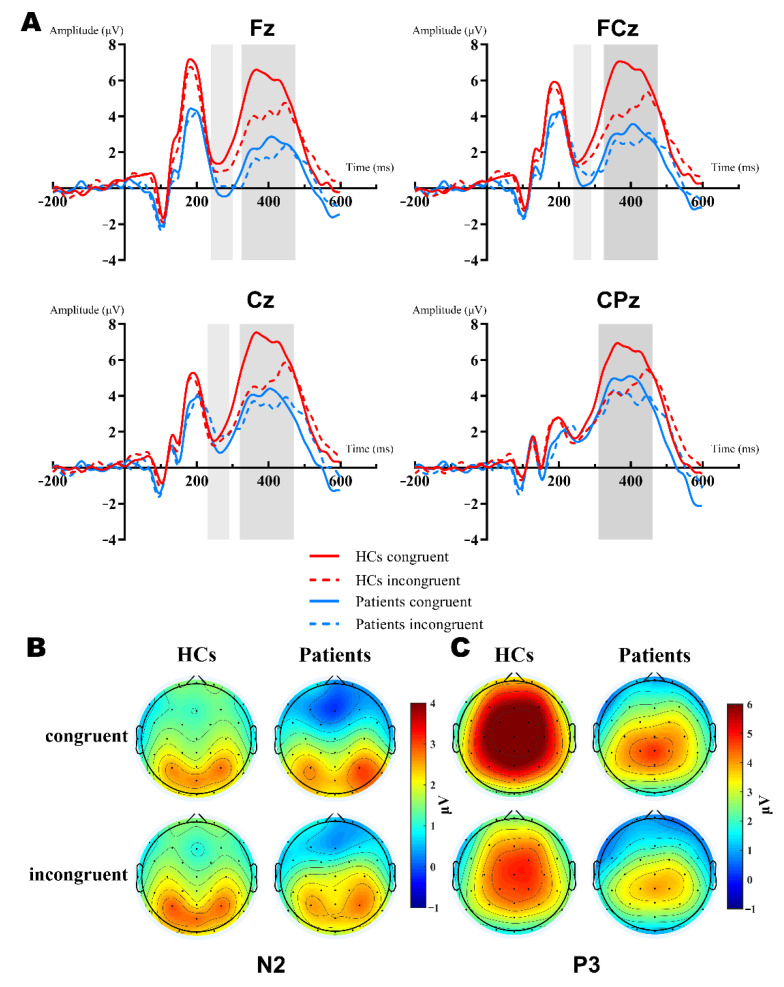
Grand-averaged ERP of the N2 and P3 waveforms and a topographic voltage map comparison of the Fz, FCz, Cz, CPz sites by group. (**A**): Averaged N2 waveform comparison of the Fz, FCz, and Cz sites (light gray background). Averaged P3 waveform comparison of the Fz, FCz, Cz, and CPz sites (dark gray background). HC group: red lines; patient group: blue lines; congruent trials: solid lines; incongruent trials: dashed lines. (**B**): N2 topographies between groups and types. (**C**): P3 topographies between groups and types.

Figure 2 showed the grand-average waveforms over frontal electrodes and scalp topography of the P2 component. Figure 3A illustrates the N2 and P3 grand-average waveforms, Figure 3B,C depicts the scalp topography of N2 and P3, separately. The statistical results of the ERP components between groups are listed next.

***P2.*** The 2 (group: HCs, patients) × 2 (type: congruent, incongruent) × 3 (site: FPz, Fz, Cz) RM-ANOVAs revealed a significant main site effect [F (2,102) = 5.343, *p* = 0.02, $\eta_{p}^{\;2}$ = 0.095], group effect [F (2,102) = 6.365, *p* = 0.015, $\eta_{p}^{\;2}$ = 0.111], and group × site interaction [F (2,102) = 7.203, *p* = 0.007, $\eta_{p}^{\;2}$ = 0.124]. Further simple effect analysis showed that there were significant differences in the frontal region (FPz, Fz), but no differences in the central region (Cz, *p* = 0.237). The P2 amplitudes of the HCs were more positive in the frontal region. FPz: HCs (M = 6.954 μV) vs. patients (M = 3.132 μV) [F (1,51) = 10.064, *p* = 0.003, $\eta_{p}^{\;2}$ = 0.165]; Fz: HCs (M = 6.35 μV) vs. patients (M = 3.833 μV) [F (1,51) = 6.568, *p* = 0.013, $\eta_{p}^{\;2}$ = 0.114]. However, both the type and type × group interactions did not reach significant differences (Figure 2).

***N2.*** The 2 (group: HCs, patients) × 2 (type: congruent, incongruent) × 3 (site: Fz, FCz, Cz) RM-ANOVA analyses of the N2 peak amplitudes revealed a significant main effect of site [F (2,102) = 15.09, *p* < 0.001, $\eta_{p}^{\;2}$ = 0.228, group [F (1,51) = 5.83, *p* = 0.019, $\eta_{p}^{\;2}$ = 0.103] and group × site interaction [F (2,102) = 4.383, *p* = 0.035, $\eta_{p}^{\;2}$ = 0.079]. Further simple effect analysis showed that there were significant differences in the frontal region (Fz, FCz), but no differences in the central region (Cz, *p* = 0.077). The N2 amplitudes of patients were more negative in the frontal-central region, Fz: HCs (M = −0.16 μV) vs. patients (M = −2.234 μV) [F (1,51) = 7.941, *p* = 0.007, $\eta_{p}^{\;2}$ = 0.135], FCz: HCs (M = 0.221 μV) vs. patients (M = −1.428 μV) [F (1,51) = 5.799, *p* = 0.02, $\eta_{p}^{\;2}$ = 0.102]. In the HCs, the N2 amplitude was more negative in the incongruent condition (M = 0.477 μV vs. −0.342 μV), while it was the opposite for patients (M = −1.833 μV vs. −1.349 μV). However, both type and type × group interaction did not reach a significant difference (Figure 3A,B). 

***P3.*** The 2 (group: HCs, patients) × 2 (type: congruent, incongruent) × 4 (site: Fz, FCz, Cz, CPz) RM-ANOVA results of the P3 mean amplitudes were quite complex. First, there was a main effect of the group [F (1,51) = 10.569, *p* = 0.002, $\eta_{p}^{\;2}$ = 0.172], type [F (1,51) = 30.363, *p* < 0.001, $\eta_{p}^{\;2}$ = 0.373], and interaction between them [F (1,51) = 30.363, *p* < 0.001, $\eta_{p}^{\;2}$ = 0.247]. Furthermore, we conducted the simple effect analysis of the group × type. Results showed that patients elicited reduced P3 amplitudes relative to the HCs in both congruent (M = 6.298 μV vs. 3.178 μV, *p* < 0.001, $\eta_{p}^{\;2}$ = 0.247) and incongruent (M = 4.417 μV vs. 2.899 μV, *p* = 0.039, $\eta_{p}^{\;2}$ = 0.081) conditions. Secondly, the results also manifested in the main effect of site [F (3,153) = 24.85, *p* < 0.001, $\eta_{p}^{\;2}$ = 0.328] and the group × site interaction [F (3,153) = 9.431, *p* = 0.001, $\eta_{p}^{\;2}$ = 0.156]. Post hoc analysis found that the P3 amplitude of the HCs were significantly more positive than patients in Fz, FCz, and Cz, except for CPz [Mean Difference (congruent—incongruent): 3.058 μV, 2.715 μV, 2.217 μV, respectively; all *p* < 0.005] (Figure 3A,C). 

A summary of the above differences in the behavioral and ERP amplitudes between the two groups is listed in Table 2.

### 3.3. Correlation Analysis Results

***PRL level and behavioral performance.*** In the patient group, Pearson correlation analyses were conducted between the PRL level, accuracy, and RT. Results showed no significant correlation coefficient between the PRL level and accuracy (both types, all *p* > 0.05), as well as between the PRL level and RT in the congruent condition (*p* > 0.05). However, RT in the incongruent condition was significant correlated with the PRL level (r = 0.426, *p* = 0.03). Hence, hyperprolactinemia was correlated with longer RTs among patients in the high-conflict condition.

**Figure 4:**
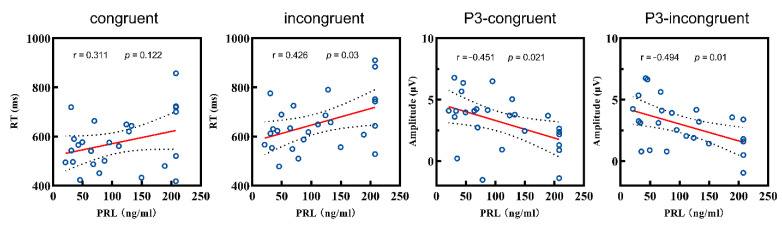
Correlations between the RT and P3 amplitudes and the PRL level of patient group. RT: reaction time; PRL: prolactin.

***PRL level and ERP.*** Based on behavioral performance, we conducted a further analysis between the PRL level and amplitudes of the ERP components (P2, N2, P3). Firstly, there was no correlation between the P2 amplitude and PRL levels under either condition (both *p* > 0.05). Secondly, the N2 amplitude was also not correlated with the PRL levels in the congruent condition (*p* > 0.05), but the correlation was marginally significant in the incongruent condition (r = −0.366, *p* = 0.066). As for the P3 analysis, the PRL level showed negative correlations with the amplitudes in both the congruent (r = −0.451, *p* = 0.021) and incongruent conditions (r = −0.494, *p* = 0.01) (Figure 4).

## 4. Discussion

This study aimed to investigate the behavioral and electrophysiological indices of attentional processing and interference control, as well as the association of these indices with the PRL level in patients with prolactinomas, using the EEG-modified Eriksen flanker task. The results provided new evidence for a potential mechanism of the persistent deleterious effects of hyperprolactinemia on cognitive function. From the perspective of behavioral performance, both groups performed with similar accuracy in both of the flanker conditions. In particular, the patient group responded more slowly to the flanker arrows than the HC group overall, but the significant difference was only manifested in the incongruent stimuli with high conflict; the difference was not reflected in congruent stimuli. From an ERP perspective, the N2 and P3 components revealed significant impairment of interference control in patients. In the P3 component analysis, remarkably reduced mean amplitudes in the patient group relative to the HC group were manifested in both the congruent and incongruent trials. As for the N2 component, more negative amplitudes were elicited in the patient group rather than the HC group. Moreover, the P2 component, which reflects the early automatic processing activity, exhibited significantly decreased mean amplitudes in the patient group relative to the HC group. The behavioral RT and P3 amplitudes correlated with the PRL level, thus further suggesting a correspondence between interference control and hormone hypersecretion. The above results are discussed in detail in the following sections.

In terms of behavioral results, both groups showed a robust main effect of the flanker types, that is, participants had better accuracy and shorter RTs in congruent trials compared with incongruent trials, suggesting that the flanker task was effective in eliciting the interference control process under a conflict situation. Patients performed with a similar accuracy to the HCs, suggesting that the task may be relatively simple. Compared with several previous flanker studies, which focused on error-related negativity (ERN) and error positivity (Pe) components, the longer inter-stimulus interval (ISI) in this study allowed sufficient time for the patients to adapt [36,37]. Nevertheless, when patients were under incongruent trials, the longer RT was a larger interference that they suffered, suggesting a disadvantage in their interference control. This group difference was unique to the incongruent trials. Combined with the accuracy result, we believe that the patients needed to spend more time completing the reaction process under the demand to achieve the same high accuracy rate as the HCs [37]. Therefore, we believed that the prolonged RT was reliable behavioral evidence of the impairment of interference control in patients.

The P2 mean amplitudes were reduced in the patient group compared with the HC group under both flanker types, whereas there was no difference in the amplitudes between the flanker types. This was a novel finding that was not quite consistent with previous results from similar studies. Indeed, the P2 component was less studied and there were still some controversies about its neural mechanism and the cognitive process that it reflects [38]. Several examples of evidence [39] suggest that the negative emotional experiences were automatically aroused by negative pictures, which were interpreted as discrepancies from the P2 modulation in the early emotion evaluation stage. The implicit emotional task elicited a frontal P2 in all conditions, indicating a rapid detection of typical stimulus features [40]. Unlike our results, previous studies of emotional stimuli paradigms in some diseases with attentional deficits (HIV, ADHD, et al.) have shown increased P2 amplitudes compared to the HCs [41]. These were interpreted as an enhanced modulation of P2 by negative stimuli and increased attention resources in compensation in the disease status [42]. However, consistent with our findings, the P2 amplitudes in the HCs in the non-emotional stimuli paradigms were significantly higher than that of the participants with attention deficits (schizophrenia [43], traumatic brain injury [44], and burnout [45]). Therefore, we believe that the differential results of the relationship between the P2 amplitudes and attention deficits can be reasonably speculated to be caused by the different stimuli paradigms and the different disease states, which need to be further explored in the future. On the other hand, Gajewski et al. [46] demonstrated that the P2 amplitudes may be modulated by stimulus consistency. However, in this study, the discriminability of relevant and irrelevant stimuli features in the arrow flanker task was high, so participants did not need to allocate significantly different attention resources for the discrimination of stimuli [47]. Taken together, reduced P2 amplitudes may indicate impaired attentional processing during early information encoding in the patient group.

Substantial evidence has confirmed that N2 and P3 components play an irreplaceable role in the ERP study of conflict resolution. N2 is considered to reflect the detection of and adaptation to mismatch and conflict, while the following P3 represents the recruitment and resource allocation necessary for task performance. Generally, N2 latency is thought to index the timing of processes engaged during decision-making, and a shorter latency is associated with a faster stimulus recognition ability [48]. In the current study, we failed to observe differences in the latency between the two groups, suggesting an unimpaired or unmanifested recognition speed in patients under the uncomplex arrow stimuli task. Regarding the N2 amplitude, the enhanced peak was usually modulated by the higher conflict [49]. However, no difference in amplitudes between the high stimulus conflict in incongruent trials and low conflict in congruent trials for either group was found. These results may be consistent with the view that the interference has been reduced under conditions of frequent cognitive conflict, such as the probabilities used in our task [38]. It has been proposed that the Go/NoGo task may be better than a flanker task in inducing a more obvious conflict detection and adaptation [29]. Nevertheless, differences in N2 amplitudes between the groups were revealed in frontal region (Fz, FCz). Previous studies [50] demonstrated that larger N2 amplitudes would be elicited when participants paid more attention to the interference arrows than the target arrow. Thus, enhanced N2 amplitudes reflected that the patients were more disturbed by the flanker congruency effect in the process of conflict resolution, and more resources were allocated for conflict monitoring and adaptation [37]. Similar characteristic deficits in interference control have already been revealed in other diseases. In high trait anxiety individuals, Righi et al. [51] observed a trend of increased N2 amplitudes, reflecting compensatory activation to conflict stimuli. 

In terms of the P3 component, the results demonstrated that P3 amplitudes over the midline sites (Fz, FCz, Cz) manifested a significant flanker congruency effect. The incongruent trials elicited lower positive-going waves compared with the congruent trials in both groups. This could be interpreted using Polich’s [32] theory, where the P3 component was regarded as a reflection of inhibition, and the number of attentional resources allocated to the inhibition process was negatively related to the P3’s amplitude. In other words, smaller P3 amplitudes meant more resources for the incongruent trials, as it generally required more resources for the evaluation and response. Conversely, under congruent conditions with lower task demands, higher synchronization activities of brain resources elicited higher P3 amplitudes [52]. The research on meditators has already confirmed these findings from the contrary perspective [53]. The meditator elicited a significantly increased P3 amplitude and no flanking effect after meditation, indicating that meditation can improve the ability to effectively allocate attention resources in tasks; more attention resources were allocated to the central target stimulus, and less to the interference stimuli. Under the above theoretical system, no matter what flanker types appear, patients would allocate more attention resources to the interference stimuli, which would further demonstrate the deficits in interference control [54]. Furthermore, although the behavioral prolonged RT and the reduced P3 amplitudes were not directly correlated, they could be mutually verified to an extent.

In summary, combined with the P2, N2, and P3 results, our findings provided credible and quantitative evidence for the impaired attentional processing and interference control in patients with prolactinomas. 

Furthermore, the strict inclusion criteria for patients minimized the confounding variable of tumor compression as much as possible, making the PRL level the most important variable. In a further correlation analysis, we found significant correlations between the RT, P3 amplitudes, and PRL level in the patient group. PRL levels were associated with a longer RT and lower incongruent P3 amplitudes, suggesting that the PRL levels may be responsible for interference control impairment. From the perspective of brain structure, our previous work revealed that patients with PAs manifested decreased gray matter volume in the prefrontal cortex (PFC) [26]. There was sufficient evidence that the PFC was directly related to inhibition control ability [55], and the modulation of the P3 amplitude was (Figure 3C) mainly manifested in the frontal-parietal regions (around Fz and Cz sites) in topographic maps. We suggested that this consistent association was not coincident. In addition, although there was no significant correlation between the P2 and N2 amplitudes and the PRL level, the topographic maps (Figure 2 and Figure 3B) also showed modulation in the adjacent frontal regions. At the molecular and cellular levels, studies have suggested that the normality of the PRL levels not only stabilizes the endocrine system, but also plays an important role in protecting the nervous system [56]. Overproduction of PRL may lead to a decrease in oligodendrocytes, thereby impairing the protective and supportive functions of the PFC, and ultimately damaging the cortical structure [14]. Therefore, we propose that the impairment of inhibition control may be caused by the toxic effects of PRL on the PFC. From another perspective, cognitive function improved in hyperprolactinemia patients that were treated with dopamine agonists [17], leading to us speculating that dopamine played an important role in cognitive decline in hyperprolactinemia. Excessive PRL may inversely inhibit dopamine activity [22], leading to mild cognitive impairment (interference control). On the other hand, the impulse control disorder (ICD) that occurred after long-term dopamine agonist administration in Parkinson’s disease (PD) patients suggested a potential cognitive toxicity to the chronic activation of dopamine activity [57,58]. Therefore, the inconsistent effects of dopamine agonists on cognitive function in different diseases implied that the effect may be bidirectional, which deserved further investigation.

Considering the following limitations, the results should be interpreted with caution. First, although there was no statistically significant difference in age between both groups, the potential confounding factors of cognitive decline caused by age span could not be completely excluded. Therefore, future studies should shorten the age span under the premise of ensuring the sample size. Secondly, although the sample size of this study has met the basic requirements of ERP studies, the inclusion of more patients could achieve a further stratified analysis of age and sex to obtain more valuable results. Thirdly, the 32-channel EEG acquisition device was applied, which could not meet the precision needs of further analysis regarding source localization. Therefore, only the correlation analysis between the PRL level and ERP indices were conducted, and the specific brain regions causing the impairment cannot be traced by this method. Further research needs to improve the density of EEG channels and combine it with other techniques.

## 5. Conclusions

The present study provided behavioral and electrophysiological evidence of impaired attentional processing and interference control in patients with prolactinomas and revealed their correlations to PRL levels. We propose that hyperprolactinemia may cause cognitive toxicity by impacting the prefrontal cortex of the brain, which provides new perspectives and insights for the clinical treatment of prolactinomas. Further studies on the cognitive function of prolactinomas and other subtypes of PAs are necessary and would be meaningful.

## Figures and Tables

**Table 1:** Summary of the participants’ characteristics.

	HCs Group	Patients Group	*p*
N	26	27	/
Females/Males	16/10	14/13	0.477 ^a^
Age (years) (M ± SD)	33.36 ± 11.97	36.08 ± 11.21	0.535 ^b^
Education (years) (M ± SD)	12.80 ± 2.32	11.21 ± 3.37	0.740 ^b^
Serum PRL (ng/mL) (M ± SD)	/	107.41 ± 68.70 Range (21.02–208)	/

^a^ Chi-square test. ^b^ Two-tailed independent-samples *t*-test. PRL: prolactin.

**Table 2:** Summary of differences in behavioral and ERP amplitudes between the two groups.

Dependent Variables	Hyperprolactinemia-Related Decrease	No Difference	Hyperprolactinemia-Related Increase
*Behavioral*			
Reaction Time			√
Accuracy		√	
*ERP*			
P2 Amplitude	√		
N2 Amplitude			√
P3 Amplitude	√		

## Data Availability

The data presented in this study are available on request from the corresponding author. The data are not publicly available due to privacy restrictions.
